# Synergistic Immunostimulatory Activities of Probiotic Strains, *Leuconostoc lactis* and *Weissella cibaria*, and the Prebiotic Oligosaccharides They Produce

**DOI:** 10.3390/microorganisms11051354

**Published:** 2023-05-22

**Authors:** Seoyoung Jeong, Ayeon Kwon, Huijin Jeong, Young-Seo Park

**Affiliations:** Department of Food Science and Biotechnology, Gachon University, Seongnam 13120, Republic of Korea; seo980914@gmail.com (S.J.); kkayeon96@gmail.com (A.K.); huijin0218@gmail.com (H.J.)

**Keywords:** probiotics, prebiotics, synbiotics, immunostimulatory activity, lactic acid bacteria, *Leuconostoc lactis*, *Weissella cibaria*, oligosaccharides

## Abstract

Synbiotics contain health-beneficial bacteria, i.e., probiotics and prebiotics selectively utilized by the probiotics. Herein, three probiotic strains, *Leuconostoc lactis* CCK940, *L. lactis* SBC001, and *Weissella cibaria* YRK005, and the oligosaccharides produced by these strains (CCK, SBC, and YRK, respectively) were used to prepare nine synbiotic combinations. Macrophages (RAW 264.7) were treated with these synbiotic combinations and the corresponding lactic acid bacteria and oligosaccharides alone to evaluate the treatments’ immunostimulatory activities. The level of nitric oxide (NO) production was significantly higher in the macrophages treated with the synbiotics than in those treated with the corresponding probiotic strains and the oligosaccharide alone. The immunostimulatory activities of the synbiotics increased regardless of the probiotic strain and the type of oligosaccharide used. The expressions of tissue necrosis factor-α, interleukin-1β, cyclooxygenase-2, inducible NO synthase genes, and extracellular-signal-regulated and c-Jun N-terminal kinases were significantly higher in the macrophages treated with the three synbiotics than in those treated with the corresponding strains or with the oligosaccharides alone. These results indicate that the synergistic immunostimulatory activities of probiotics and the prebiotics they produced in the studied synbiotic preparations resulted from the activation of the mitogen-activated protein-kinase-signaling pathway. This study suggests the combined use of these probiotics and prebiotics in the development of synbiotic preparations as health supplements.

## 1. Introduction

Probiotics are beneficial for host health and balance the intestinal flora of the host [1]. They are defined by the Food and Agriculture Organization/World Health Organization as “live microorganisms that, when administrated in adequate amounts, confer health benefits to the host” [2]. Probiotics have many positive effects on health, and they have been broadly studied and examined commercially in many products [3]. Recently, probiotics have primarily been used for improving gastrointestinal health, and they can be easily applied to improve oral, skin, and vaginal health [4]. Their beneficial effects on gastrointestinal health include their balancing of the intestinal microflora, immunomodulation, the enhancement of absorption and digestion, the synthesis of vitamins, the inhibition of the growth of potential pathogens, and reductions in cholesterol levels [5].

Oligosaccharides are compounds with 2–10 degrees of polymerization (DP), and they are categorized based on their DP and chemical characteristics [6,7]. Furthermore, they are the primary sources of carbohydrates for lactic acid bacteria and act as prebiotics [6]. Prebiotics are defined by the International Scientific Association for Probiotics and Prebiotics (ISAPP) as “substrates that are selectively utilized by host microorganisms, conferring health benefits” [8]. They include fructo-oligosaccharides (FOS), galacto-oligosaccharides (GOS), xylo-oligosaccharides (XOS), and inulin [9]. Prebiotics are extracted from plants or produced via enzymatic reactions. Gluco-oligosaccharides are produced through the cleavage of polymers by intestinal microflora. Prebiotics are produced by *Lactobacillus, Leuconostoc, Weissella*, and *Streptococcus* species, which contain enzymes such as alternating sucrase (EC 2.4.1.140), glucansucrase (EC 2.4.1.5), levansucrase (EC 2.4.1.10), and mutansucrase (EC 2.4.1.5), which catalyze the synthesis of dextrans (glucans) [10,11,12,13,14]. Inulin-type prebiotic compounds have been detected in many plants, particularly chicory (*Cichorium intybus*) [15,16].

The term synbiotic was defined by ISAPP, in 2020, as a mix of live microorganisms and substrate(s) selectively utilized by host microorganisms that confer health benefits to the host [17]. Prebiotics, which are specific probiotic substrates, can be added to probiotics to prepare synbiotics (e.g., lactitol + lactobacilli or FOS + bifidobacterial strains). Synbiotics manufactured in this manner assist in the survival and growth of probiotic organisms by fermenting specific substrates. Therefore, it is possible to derive health benefits from living microorganisms and prebiotics [18]. Synbiotics exhibit various health benefits and therapeutic applications. They are effective against diseases including diarrhea, inflammatory bowel disease, irritable bowel syndrome, childhood infections, lactose intolerance, cancer, trauma, liver diseases, allergic asthma, and diabetes [19].

Based on their specificity and speed of reaction, immune responses are divided into innate and adaptive, and several interactions occur between them [20]. As the first lines of defense, early innate immune responses are crucial for controlling the initial infection of naive cells [21]. Higher biological immune systems, including antigen-specific responses by T cells and B lymphocytes, are characterized as adaptive immune responses [22]. Inflammatory monocytes, such as murine Ly6c^+^, are drawn from circulation after tissue damage or infection and move into afflicted tissues, where they develop into macrophages [23]. Macrophages are immune cells that are essential for both innate and adaptive immunological responses. The macrophages involved in innate and adaptive immune responses serve as the first lines of defense against invasion by foreign pathogens and regulate leukocyte infiltration [24]. In the initial phases of the immune response, these differentiated macrophages frequently release various inflammatory mediators, such as tissue necrosis factor (TNF)-α, interleukin (IL)-1, and nitric oxide (NO), and exhibit pro-inflammatory responses [23]. Small proteins called cytokines are crucial for cell signaling in the immune system. Produced and released by cells, cytokines influence the behavior and production of other cells. There are many different types of cytokine, such as TNF-α, IL, interferon (IFN), lymphokines, and chemokines [25]. A primary characteristic of probiotics is their ability to modulate the levels of anti-inflammatory cytokines, such as IL-10 and transforming growth factor-β, and pro-inflammatory cytokines, such as IL-1, IL-6, IL-12, TNF-α, and IFN-γ [26,27,28]. Antigen presentation and immunological stimulation activities are examples of signals that cause cytokine production. In general, monocytes, lymphocytes, and macrophages are the principal producers of cytokines and can swiftly generate cytokines once activated; this in turn, affects the immune system in humans [29,30].

In our previous studies, oligosaccharide-producing strains of lactic acid bacteria *Leuconostoc lactis* CCK940, *L. lactis* SBC001, and *Weissella cibaria* YRK005 were isolated, and the prebiotic, immunostimulatory, and anti-inflammatory activities of the oligosaccharides produced were investigated [31,32,33]. It was shown that the oligosaccharides produced by *L. lactis* CCK940 and *W. cibaria* YRK005 have immunostimulatory activities, and that those generated by *L. lactis* SBC001 have anti-inflammatory activities [32,34,35].

We examined the probiotic characteristics of the *L. lactis* CCK940, *L. lactis* SBC001, and *W. cibaria* YRK005 strains and investigated the synergistic immunostimulatory activities of the synbiotics prepared by combining these probiotic lactic acid bacteria with their corresponding prebiotic oligosaccharides.

## 2. Materials and Methods

### 2.1. Culture of Lactic Acid Bacteria

Lactic acid bacterial strains used in this study were *L. lactis* CCK940 (KCCM 11724P) isolated from Chinese cabbage kimchi [31], *L. lactis* SBC001 (KCCM 43402) isolated from [32], and *W. cibaria* YRK005 isolated from young radish kimchi (KCCM 42404) [33].

Lactic acid bacterial strains were cultured on de Man–Rogosa–Sharpe medium (MRS; Becton Dickinson and Company, Franklin Lakes, NJ, USA) at 37 °C for 16 h. The MRS medium consisted of 10.0, 10.0, 5.0, 20.0, 5.0, 1.0, 2.0, 2.0, 0.1, and 0.05 g of protease peptone no. 3, beef extract, yeast extract, glucose, sodium acetate, polysorbate 80, dipotassium hydrogen phosphate, ammonium citrate, magnesium sulfate, and manganese sulfate per liter of water.

### 2.2. Culture of Animal Cells

#### 2.2.1. Cell Lines and Culture Conditions

A murine macrophage cell line, RAW 264.7, was purchased from the Korean Cell Line Bank (Seoul, Republic of Korea). These cells were cultured in Dulbecco’s modified Eagle’s medium (DMEM; Gibco, Grand Island, NY, USA) supplemented with 10% (*v*/*v*) bovine serum (Gibco) and 1% penicillin–streptomycin solution (Gibco), followed by incubation at 37 °C in an incubator with 5% CO_2_ (Thermo Fisher Scientific, Waltham, MA, USA).

Human colon adenocarcinoma cell line, HT-29, was purchased from the Korean Cell Line Bank and cultured in DMEM supplemented with 10% fetal bovine serum (FBS; Corning Inc., Corning, NY, USA) and 1% (*v*/*v*) penicillin–streptomycin solution, followed by incubation at 37 °C in an incubator with 5% CO_2_. The cells were detached from the culture flask using 0.25% trypsin–ethylenediaminetetraacetic acid (Gibco).

#### 2.2.2. Preparation of Lactic Acid Bacterial Sample

The MRS broth was inoculated with the culture broth containing lactic acid bacteria at a final concentration of 1% (*v*/*v*) and incubated at 37 °C until the cell density of each lactic acid bacterial strain was approximately 1 × 10^5^ colony-forming units (CFU)/mL. One milliliter of the culture broth was centrifuged five times at 15,920× *g* for 1 min each. The harvested pellets were washed three times with Dulbecco’s phosphate-buffered saline (DPBS, Welgene, Gyeongsan, Republic of Korea) and resuspended in DMEM containing 10% FBS and 1% penicillin–streptomycin solution. These bacterial suspensions were diluted with DMEM supplemented with 10% FBS and 1% penicillin–streptomycin solution for each multiplicity of infection (MOI; bacterial cell number/macrophage cell number), which is the ratio of viable bacterial cells to macrophages. The bacterial suspensions were diluted in DMEM supplemented with 10% FBS for the gut-adhesion assay.

### 2.3. Evaluation of Probiotic Characteristics of Lactic Acid Bacteria

#### 2.3.1. Acid Tolerance of Lactic Acid Bacteria

The overnight lactic acid bacterial culture was inoculated in MRS broth at a final concentration of 0.5% (*v*/*v*) and incubated at 37 °C for 16 h. After centrifugation of the culture broth at 21,200× *g* for 1 min, the harvested pellets were washed twice with 0.88% (*w*/*v*) sodium chloride. The washed pellets were resuspended in 100 mM of glycine–HCl buffer and pH was adjusted to 2.5. The bacterial suspension was serially diluted with 0.88% (*w*/*v*) sodium chloride at 0 h and spread onto Petri films (3M Company, Saint Paul, MN, USA) as control samples. After the bacterial suspensions were incubated at 37 °C for 2 h, they were serially diluted with 0.88% (*w*/*v*) sodium chloride and spread onto Petri films as experimental samples. The Petri films were incubated at 37 °C for 20 h, followed by counting of the number of viable cells. Acid tolerance was calculated as follows:

(1) $$\mathrm{Acid}\;\mathrm{tolerance}\;(\%)=\frac{\log\left(\mathrm{Viable}\;\mathrm{cell}\;\mathrm{number}\;\mathrm{after}\;2\;h\;\left(\mathrm{sample}\right)\right)}{\log\left(\mathrm{Viable}\;\mathrm{cell}\;\mathrm{number}\;\mathrm{after}\;0\;h\;\left(\mathrm{control}\right)\right)}\times 100\;(\%).$$

#### 2.3.2. Bile Tolerance

The overnight culture of lactic acid bacteria was inoculated into MRS broth, with or without 0.3% (*w*/*v*) Bacto–Oxgall media (Becton Dickinson and Company), at a final concentration of 0.5% (*v*/*v*) and incubated at 37 °C for 16 h. After incubation, the bacterial suspension was serially diluted with 0.88% (*w*/*v*) sodium chloride and spread onto Petri dishes. The Petri films were incubated at 37 °C for 20 h, followed by counting of the number of viable cells. Bile tolerance was calculated as follows.

(2) $$\mathrm{Bile}\;\mathrm{tolerance}\;(\%)=\frac{\log\left(\mathrm{Viable}\;\mathrm{cell}\;\mathrm{number}\;\mathrm{in}\;\mathrm{the}\;\mathrm{presence}\;\mathrm{of}\;\mathrm{Oxgall}\right)}{\log(\mathrm{Viable}\;\mathrm{cell}\;\mathrm{number}\;\mathrm{in}\;\mathrm{the}\;\mathrm{absence}\;\mathrm{of}\;\mathrm{Oxgall})}\times 100\;(\%).$$

#### 2.3.3. Gut-Adhesion Ability in an In Vitro Model 

The HT-29 cells (5 × 10^5^ cells) were plated in each well of a 24-well plate, incubated at 37 °C for 20 h, treated with lactic acid bacteria (1 × 10^5^ CFU/mL), and suspended in DMEM supplemented with 10% FBS but without antibiotics, as mentioned in Section 2.2.2. The 24-well plate with the cultured HT-29 cells treated with lactic acid bacteria was incubated at 37 °C with 5% CO_2_ for 2 h for the cells to adhere. Lactic acid bacteria (1 × 10^8^ CFU/mL) resuspended in antibiotic-free DMEM and incubated at 37 °C for 2 h were used in a control sample. The culture medium in the 24-well plate was removed, and cell monolayers were washed four times with DPBS to remove non-adherent bacterial cells. After 200 μL of 0.25% trypsin–EDTA was added to each well, the plate was incubated at 37 °C with 5% CO_2_ for 5 min. The cells were collected and transferred to new tubes. Next, they were washed twice with DPBS at 21,200× *g* for 1 min each and treated with 0.1% (*v*/*v*) Triton X-100 in DPBS. Experimental and control samples were spread onto Petri films and incubated at 37 °C for 16 h, followed by counting of the number of viable cells. The percentage ratio of the adhesion ability was calculated as follows:

(3) $$\mathrm{Gut}\;\mathrm{adhesion}\;\mathrm{ability}\;(\%)=\frac{\log\left(\mathrm{Viable}\;\mathrm{cell}\;\mathrm{number}\;\mathrm{of}\;\mathrm{sample}\right)}{\log\left(\mathrm{Viable}\;\mathrm{cell}\;\mathrm{number}\;\mathrm{of}\;\mathrm{control}\right)}\times 100\;(\%).$$

#### 2.3.4. Antioxidant Activity of Lactic Acid Bacteria

A 2,2-diphenyl-1-picrylhydrazyl (DPPH; Sigma-Aldrich, St. Louis, MO, USA) radical-scavenging assay was performed to determine the radical-scavenging activity of probiotic lactic acid bacteria. Ascorbic acid was used at 0–2-mM concentrations to generate a standard curve. The 2.0-mM concentration of ascorbic acid was used as the positive control, and deionized water was used as the negative control. The overnight lactic acid bacterial culture was inoculated in MRS broth at a final concentration of 1% (*v*/*v*) and incubated at 37 °C for 16 h. After centrifuging the culture at 21,200× *g* for 5 min, 10.0 μL of culture supernatant was transferred to a 96-well plate. Approximately 190 μL of 0.1 mM DPPH was added to each well of the 96-well plate and incubated at 20 °C for 30 min. The DPPH’s scavenging activity was monitored by measuring the absorbance at 517 nm using a microplate reader (Epoch microplate reader; BioTek Instruments Inc., Winooski, VT, USA). The DPPH’s radical-scavenging activity was calculated as follows:

(4) $$\mathrm{DPPH}\;\mathrm{radical}\text{-}\mathrm{scavenging}\;\mathrm{activity}\;(\%)=\left(1-\frac{A_{517,\;\mathrm{sample}}}{A_{517,\;\mathrm{negative}\;\mathrm{control}}}\right)\times 100\;\left(\%\right).$$

### 2.4. Preparation of Oligosaccharides

The oligosaccharides produced by *L. lactis* CCK940, *L. lactis* SBC001, and *W. cibaria* YRK005 strains were purified as previously described [11,32,33]. In this study, the oligosaccharides produced by *L. lactis* CCK940, *L. lactis* SBC001, and *W. cibaria* YRK005 strains were named CCK-, SBC-, and YRK-oligosaccharides, respectively. In brief, the culture supernatant of lactic acid bacteria was obtained by centrifuging at 9820× *g* for 15 min (Beckman Coulter, Brea, CA, USA), and the supernatant was concentrated under reduced pressure at 60 °C. The concentrated supernatant was then loaded onto Bio-Gel P2 in a Glass Econo-Column (1.5 × 120 cm), and the oligosaccharides were separated by gel-permeation chromatography (GPC) (Bio-Rad, Hercules, CA, USA). The flow rate was 0.5 mL/min, and the eluents were fractionated using a fraction collector (Gilson Inc., Middleton, WI, USA) with 5 mL per tube. The fractions were analyzed by TLC, and the active fractions containing oligosaccharides were collected and lyophilized (SunilEyela, Seongnam, Republic of Korea).

### 2.5. Preparation of Synbiotics Containing Oligosaccharide-Producing Probiotic Strains and Their Corresponding Oligosaccharides

Nine synbiotics were prepared using three prebiotic oligosaccharides (CCK-, SBC-, and YRK-oligosaccharides) and the corresponding probiotic lactic acid bacterial strains (*L. lactis* CCK940, *L. lactis* SBC001, and *W. cibaria* YRK005, respectively) producing these oligosaccharides (Table 1). The oligosaccharide concentrations and MOIs of lactic acid bacteria used for treating macrophages were determined based on previous studies and preliminary experiments [34,35]. Macrophages were treated with CCK-oligosaccharides and SBC-oligosaccharides at concentrations of 0.25 mg/mL each and with YRK-oligosaccharides at a concentration of 0.1 mg/mL. Similarly, macrophages were treated with *L. lactis* CCK940 strain at an MOI of 10 and with *L. lactis* SBC001 and *W. cibaria* YRK005 strains at MOI of 20 each. 

### 2.6. Evaluation of Immunostimulatory Activities

#### 2.6.1. Cell-Viability Assay

The cell viability of RAW 264.7 macrophages was checked using the EZ-CYTOX kit (DoGenBio, Seoul, Republic of Korea). The RAW 264.7 cells were plated at a cell density of 5×10^4^ cells in each well of a 96-well plate and incubated at 37 °C for 20 h. Next, these cells were treated with lactic acid bacteria to obtain an MOI of 0, 0.1, 1, 10, 100, and 500 and incubated at 37 °C for 24 h. A MOI of 0 was used as a control. The cultured wells were washed three times with DPBS, and 200 μL of DMEM supplemented with 10% (*v*/*v*) bovine serum and 1% (*v*/*v*) penicillin–streptomycin was added. Next, 20 μL of EZ-CYTOX reagent was added and incubated at 37 °C for 30 min. The viability of RAW 264.7 cells was calculated by measuring the absorbance at a wavelength of 450 nm using the microplate reader (Epoch microplate reader; BioTek Instruments Inc.) as follows:

(5) $$\mathrm{Cell}\;\mathrm{viability}\;(\%)=\left(\frac{A_{\;\mathrm{sample}}}{A_{\;\mathrm{control}}}\right)\times 100\;(\%).$$

#### 2.6.2. Nitric Oxide Assay

The RAW 264.7 cells were plated at a density of 5 × 10^5^ cells in each well of a 24-well plate and incubated at 37 °C for 20 h. The macrophages were treated with the CCK-, SBC-, and YRK-oligosaccharides and with the cultured lactic acid bacteria at each indicated concentration (Section 2.5) and incubated at 37 °C for 24 h. The RAW 264.7 cells treated with 1 μg/mL lipopolysaccharides (LPS) (cat. no. L4391, Sigma-Aldrich) at 37 °C for 24 h were used as positive controls. An MOI of 0 was used as a negative control. Next, 100 μL of the culture supernatant was transferred onto a 96-well plate, mixed with 100 μL of Griess reagent (reagent A: 0.1% *N*-(1-naphthyl)-ethylenediamine dihydrochloride; reagent B: 2.5% phosphoric acid containing 1% sulfanilamide) (Sigma-Aldrich), and allowed to react at 20 °C for 15 min. The amount of NO produced by the activated macrophages was estimated by measuring the absorbance at a wavelength of 540 nm using the microplate reader. The 0–250-μM concentrations of sodium nitrite in DMEM were used to generate a standard curve. In addition, a NO assay for synbiotics was conducted using nine synbiotic combinations (Table 1).

#### 2.6.3. Total-RNA Extraction and the Synthesis of cDNA

The RAW 264.7 cells were plated at a cell density of 1 × 10^6^ cells in each well of a 6-well plate and incubated at 37 °C for 20 h. Macrophages were treated with the CCK-, SBC-, and YRK-oligosaccharides and with lactic acid bacteria at the indicated concentrations (Section 2.5) and incubated at 37 °C for 24 h. The RAW 264.7 cells were treated with 1 μg/mL LPS at 37 °C for 24 h as positive controls. A MOI of 0 was used as a negative control. After incubation, the activated cells were washed twice with DPBS, and an easy-BLUE^TM^ Total RNA Extraction Kit (iNtRON Biotechnology Inc., Seongnam, Republic of Korea) was employed to extract RNA from the activated cells. After RNA extraction, cDNA was synthesized from the extracted RNA using CellScript^TM^ cDNA Master Mix (CellSafe, Suwon, Republic of Korea).

#### 2.6.4. Real-Time Quantitative Polymerase Chain Reaction (PCR)

Real-time quantitative PCR (qPCR) was performed to analyze the expression levels of cytokine mRNAs relative to *TNF-α*, *IL-1β*, inducible nitric oxide synthase (*iNOS*), and cyclooxygenase-2 (*COX-2*) genes. The expression levels of cytokine mRNAs were determined using the delta–delta Ct, i.e., 2^−ddCT^, a method that uses the expression levels of glyceraldehyde-3-phosphate dehydrogenase, i.e., the *GAPDH* gene, as an endogenous control [36]. The qPCR was performed using the LightCycler^®^ 96 instrument (Roche, Basel, Switzerland) and QGreenBlue^TM^ 2X Green qPCR Master Mix (CellSafe) following the manufacturer’s instructions. Primer sequences used for the cytokine genes are listed in Table 2. 

#### 2.6.5. Protein Extraction

The methods used to treat the experimental samples on the cell surface for protein extraction were the same as those described in Section 2.6.3. After incubation, the activated cells were washed twice with cold DPBS and lysed using 200 μL of radioimmunoprecipitation-assay buffer (Cell Signaling Technology, Beverly, MA, USA) containing 1% (*v*/*v*) protease-inhibitor cocktail (GenDEPOT, Katy, TX, USA) and 1% (*v*/*v*) phosphatase-inhibitor cocktail (GenDEPOT) for cell lysis through reaction on ice for 30 min. The cell lysates were centrifuged at 14,000× *g* for 10 min at 4 °C, the supernatants were harvested, and protein concentration was quantified using the Pierce^TM^ BCA protein-assay kit (Thermo Fisher Scientific).

#### 2.6.6. Western Blotting

Fifty micrograms of proteins per sample were separated by 12.5% sodium dodecyl sulfate–polyacrylamide-gel electrophoresis and transferred to nitrocellulose membranes (Bio-Rad Laboratories Inc., Hercules, CA, USA). The membranes were blocked with 3% (*w*/*v*) FBS in 100 mM Tris-buffered saline containing 0.1% (*v*/*v*) Tween 20 (TBS-T). Following the blocking step, the membranes were incubated with primary antibodies against p38, phospho-p38 (p-p38), extracellular signal-regulated kinase (ERK)1/2, phosphor-ERK1/2 (p-ERK1/2), c-Jun N-terminal kinase (JNK), phospho-JNK (p-JNK), and β-actin (1:1000, Cell Signaling Technology, Danvers, MA, USA) at 4 °C overnight. After washing thrice with TBS-T, the membranes were incubated with secondary antibodies (1:1000; anti-rabbit IgG, Cell Signaling Technology) at 20 °C for 1 h and washed thrice with TBS-T. Chemiluminescent signals emitted after incubation with secondary antibodies were detected with ECL solution (EzWestLumi plus chemiluminescent substrate; ATTO Corporation, Tokyo, Japan) using an Amersham^TM^ Imager 600 (Cytiva, Amersham, UK). Western blotting was also conducted with three of the nine synbiotic combinations, as reported in Table 1.

### 2.7. Statistical Analyses

The experiments were performed in triplicate and repeated three times. Data were expressed as mean ± standard deviation from triplicate experiments. Statistical analyses were conducted using SPSS 23 (SPSS Inc., Chicago, IL, USA). Statistical significance between multiple groups was determined using a one-way analysis of variance, followed by Duncan’s multiple-range test (*p* < 0.05).

## 3. Results

### 3.1. Probiotic Characteristics of Oligosaccharide-Producing Lactic Acid Bacteria

#### 3.1.1. Acid and Bile Tolerances and the Ability to Adhere to HT-29 Cells

As the three lactic strains were exposed to pH 2.5, the number of viable cells tended to decrease (Table 3). The numbers of viable cells in the *L. lactis* SBC001 and *W. cibaria* YRK005 strains decreased by 4.35 and 4.23 log CFU/mL, respectively, whereas that in the *L. lactis* CCK940 strain decreased by 1.16. log CFU/mL. These results indicated that the *L. lactis* CCK940, *L. lactis* SBC001, and *W. cibaria* YRK005 strains exhibited 87.2, 52.3, and 54.0% acid tolerances, respectively. As the three lactic acid bacterial strains were exposed to 0.3% Oxgall media, the counts of the viable cells decreased compared to those in the MRS media without Oxgall. However, the decreases in the counts of viable cells were lower than those under acidic conditions. The counts of viable cells in the *L. lactis* CCK940, *L. lactis* SBC001, and *W. cibaria* YRK005 strains decreased by 2.14, 1.59, and 1.43 log CFU/mL, respectively, indicating similar bile tolerances among the three lactic acid bacterial strains.

The abilities of the *L. lactis* CCK940, *L. lactis* SBC001, and *W. cibaria* YRK005 strains to adhere to the HT-29 cells were 54.2, 55.7, and 67.6%, respectively (Table 3).

#### 3.1.2. Antioxidant Activities of Oligosaccharide-Producing Lactic Acid Bacteria 

The antioxidant activities of the cell-free supernatants of the three prebiotic-producing lactic acid bacteria were examined by using a DPPH assay. The DPPH radical-scavenging activities of *L. lactis* CCK940, *L. lactis* SBC001, and *W. cibaria* YRK005 strains were 56.8, 67.1, and 64.5%, respectively (Table 4) compared with that of the 2-mM ascorbic acid used as the positive control. 

### 3.2. Immunostimulatory Activities of Prebiotic-Producing Lactic Acid Bacteria

#### 3.2.1. Cell Viability

**Figure 1:**
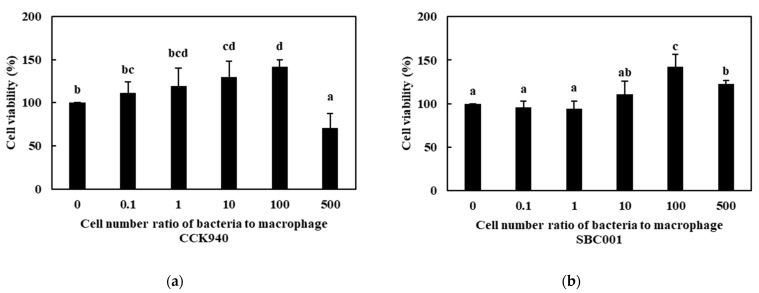

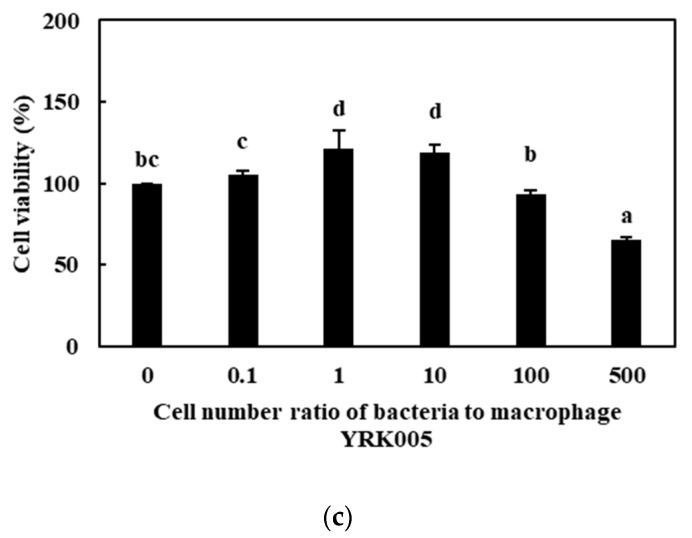
Effects of *Leuconostoc lactis* CCK940, *L. lactis* SBC001, and *Weissella cibaria* YRK005 strains on the cell viability of RAW 264.7 cells. The multiplicities of infection (MOI, bacterial cell number/macrophage cell number) were 0, 0.1, 1, 10, 100, and 500 for each strain. (**a**) *L. lactis* CCK940, (**b**) *L. lactis* SBC001, and (**c**) *W. cibaria* YRK005. Different alphabet letters between groups represent significant differences at *p* < 0.05.

The effects of the prebiotic-producing lactic acid bacteria on the viability of the RAW 264.7 cells are shown in Figure 1. The viability of the RAW 264.7 cells treated with the *L. lactis* SBC001 strain was over 100% at all the MOI concentrations, which indicated that the *L. lactis* SBC001 strain had no cytotoxic effect on the RAW 264.7 cells. In contrast, the *L. lactis* CCK940 and *W. cibaria* YRK005 strains exhibited no cytotoxic effects below MOI 100. However, the viabilities of the RAW 264.7 cells treated with the *L. lactis* CCK940 and *W. cibaria* YRK005 strains at MOI 500 were 70.8% and 65.6%, respectively, which showed that there was a considerable cytotoxic effect on the RAW 264.7 cells at MOI over 500. 

#### 3.2.2. Production of NO

**Figure 2:**
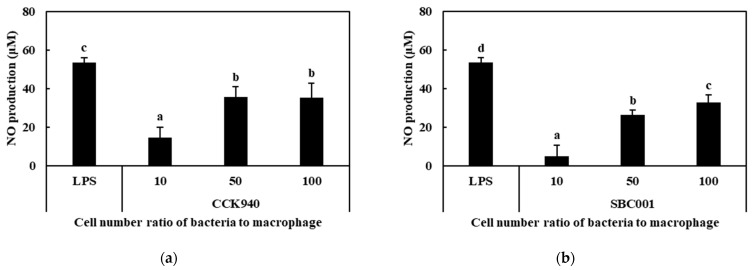

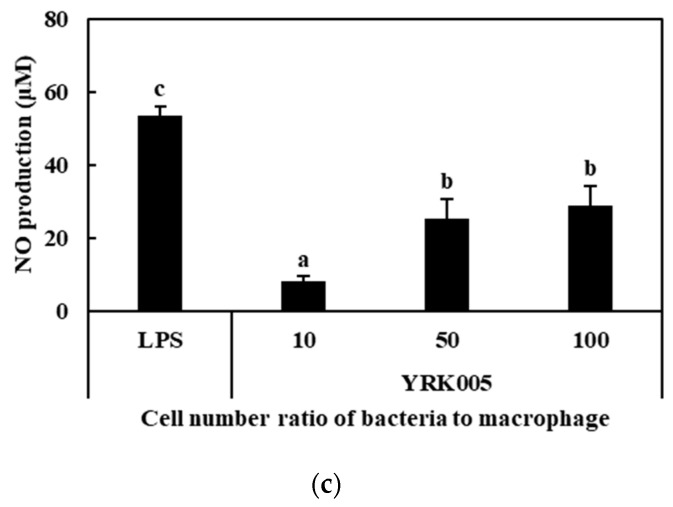
Nitric oxide (NO) production by RAW 264.7 macrophages treated with (**a**) *L. lactis* CCK940, (**b**) *L. lactis* SBC001, and (**c**) *W. cibaria* YRK005 strains. The MOI values were 10, 50, and 100 for each strain. Macrophages treated with 1 μg/mL of lipopolysaccharides (LPS) were used as the positive control sample. Different alphabet letters between groups represent significant differences at *p* < 0.05.

As the RAW 264.7 cells were treated with prebiotic-producing lactic acid bacteria at MOI concentrations of 10, 50, and 100, the NO produced by the activated macrophages increased with the increasing MOI concentrations (Figure 2). At MOI 100, the concentrations of NO produced by the macrophages treated with the *L. lactis* CCK940, *L. lactis* SBC001, and *W. cibaria* YRK005 strains were 35.3, 32.7, and 28.8 μM, respectively. The macrophages treated with 1 μg/mL of LPS produced 53.4 μM NO. These results indicates that the *L. lactis* CCK940, *L. lactis* SBC001, and *W. cibaria* YRK005 strains had immunostimulatory effects on the RAW 264.7 macrophage cells. 

#### 3.2.3. Quantitative Analysis of Cytokine Expression in Macrophages Treated with Lactic Acid Bacteria

**Figure 3:**
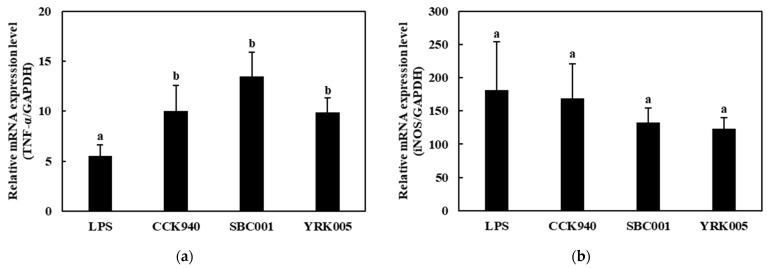

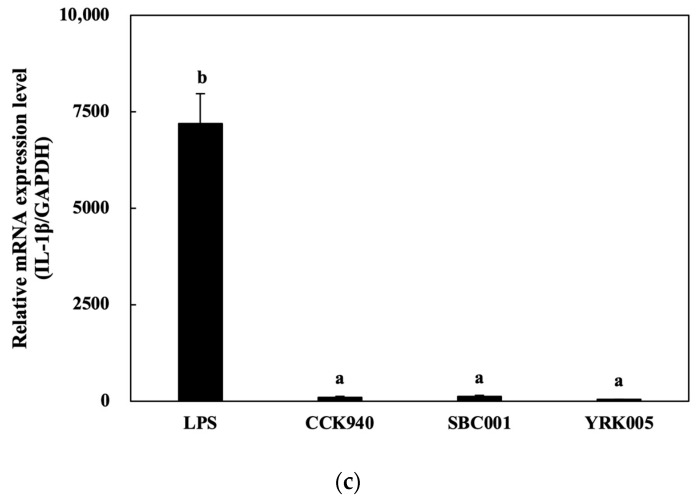
Effects of the *L. lactis* CCK940, *L. lactis* SBC001, and *W. cibaria* YRK005 strains on the mRNA-expression levels of (**a**) tissue necrosis factor-α (*TNF-α*), (**b***)* inducible nitric oxide synthase (*iNOS*), and (**c**) interleukin-1β (*IL-1β*) genes in RAW 264.7 cells. The MOI was 100 for each strain. Macrophages treated with 1 μg/mL of lipopolysaccharides (LPS) were used as the positive control sample. Different alphabet letters between groups represent significant differences at *p* < 0.05.

The mRNA expression levels of the *TNF-α* gene in the RAW 264.7 cells treated with the three lactic acid bacterial strains at MOI 100 were significantly higher than those in the positive control. The treatment with the *L. lactis* SBC001 strain induced the highest level of *TNF-α* expression (Figure 3a). The IL-1β-expression levels showed no notable differences between the macrophage samples treated with the three lactic acid bacterial strains (Figure 3b). The mRNA expression levels of the *iNOS* were higher in the macrophages treated with the *L. lactis* CCK940, *L. lactis* SBC001, and *W. cibaria* YRK005 strains than in the LPS-treated macrophages (Figure 3c). 

#### 3.2.4. Quantitative Analysis of the Expression of the Mitogen-Activated Protein Kinase (MAPK)-Pathway Proteins in Macrophages Treated with Lactic Acid Bacterial Strains

**Figure 4:**
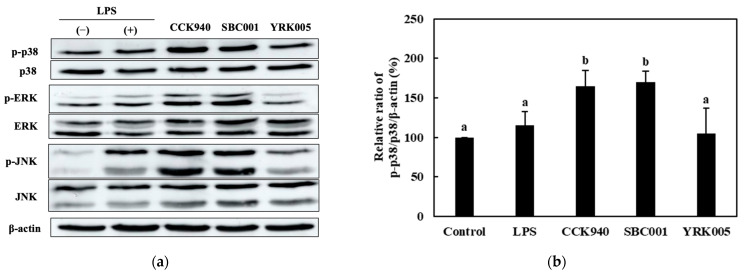

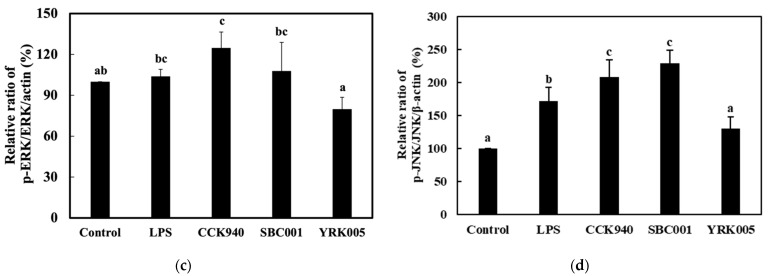
Effects of *L. lactis* CCK940, *L. lactis* SBC001, and *W. cibaria* YRK005 treatments on the mitogen-activated protein kinase (MAPK)-signaling pathway in RAW 264.7 macrophages. The MOI was 100 for each strain. (**a**) Western blot reveals the expression of MAPK-pathway proteins in macrophages treated with the three bacterial strains. Graphs depict the relative ratios of (**b**) p-p38/p38, (**c**) p-ERK/ERK, and (**d**) p-JNK/JNK in the treated macrophages. Western blot is representative of three independent experiments. Macrophages treated with 1 μg/mL of lipopolysaccharides (LPS) were used as the positive control sample. Untreated macrophages were used as the negative control sample. Different alphabet letters among groups represent significant differences at *p* < 0.05.

The RAW 264.7 macrophages treated with the *L. lactis* CCK940 and *L. lactis* SBC001 strains revealed significantly higher ratios of p-p38/p38, p-ERK/ERK, and p-JNK/JNK than the LPS-treated macrophages (Figure 4). In the macrophages treated with the *W. cibaria* YRK005 strain, the p-p38/p-38 and p-JNK/JNK ratios were significantly higher than those in the control group (Figure 4b,d), and the p-ERK/ERK ratio was significantly lower than in the control group (Figure 4c). 

### 3.3. Synergistic Immunostimulatory Activities of Probiotics and the Prebiotics They Produced 

#### 3.3.1. Production of NO

**Figure 5:**
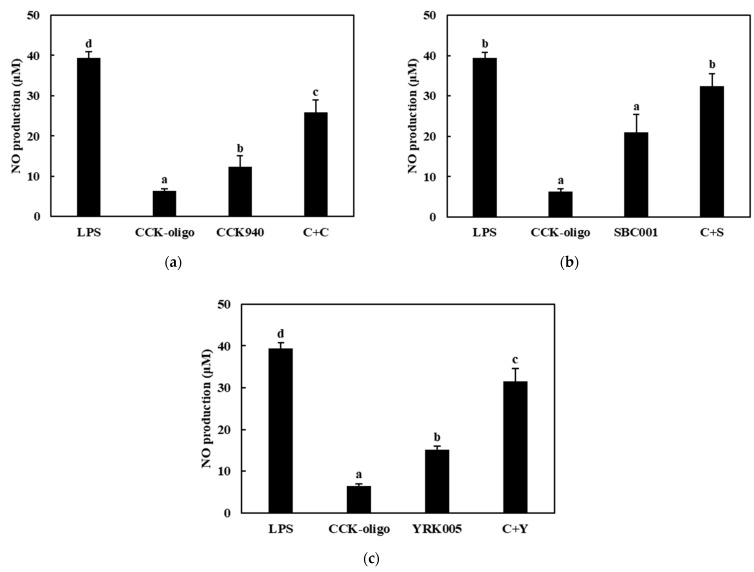
Effects of synbiotics containing CCK-oligosaccharides on nitric oxide production in RAW 264.7 macrophages. The combinations of probiotics and prebiotics used were: (**a**) CCK-oligosaccharides + *L. lactis* CCK940 (C + C): 0.25 mg/mL of CCK-oligosaccharides and MOI 10 of *L. lactis* CCK940; (**b**) CCK-oligosaccharides + *L. lactis* SBC001 (C + S): 0.25 mg/mL of CCK-oligosaccharides and MOI 20 of *L. lactis* SBC001; and (**c**) CCK-oligosaccharides + *W. cibaria* YRK005 (C + Y): 0.25 mg/mL of CCK-oligosaccharides and MOI 20 of *W. cibaria* YRK005. Macrophages treated with 1 μg/mL of lipopolysaccharides (LPS) were used as the positive control sample. Different alphabet letters between groups represent significant differences at *p* < 0.05.

**Figure 6:**
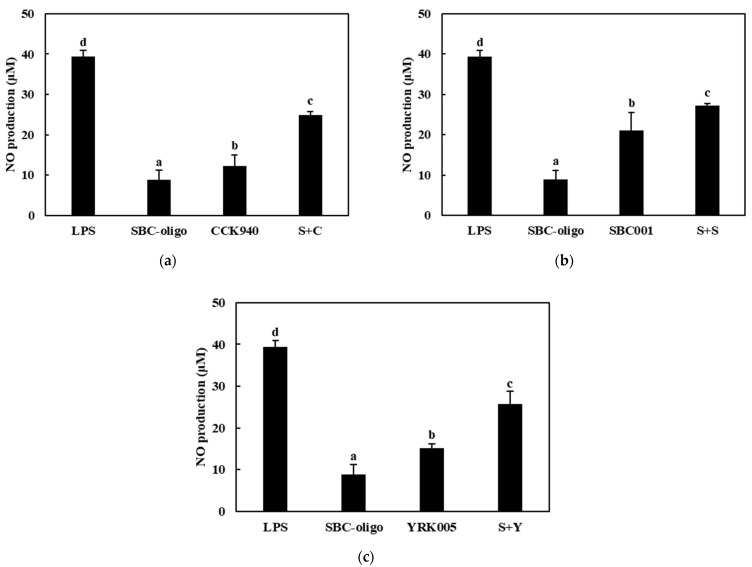
Effects of synbiotics containing SBC-oligosaccharides on nitric oxide production in RAW 264.7 macrophages. The combinations of probiotics and prebiotics used were: (**a**) SBC-oligosaccharides + *L. lactis* CCK940 (S + C): 0.1 mg/mL of SBC-oligosaccharides and MOI 10 of *L. lactis* CCK940; (**b**) SBC-oligosaccharides + *L. lactis* SBC001 (S + S): 0.1 mg/mL of SBC-oligosaccharides and MOI 20 of *L. lactis* SBC001; and (**c**) SBC-oligosaccharides + *W. cibaria* YRK005 (S + Y): 0.1 mg/mL of SBC-oligosaccharides and MOI 20 of *W. cibaria* YRK005. Macrophages treated with 1 μg/mL of lipopolysaccharides (LPS) were used as the positive control sample. Different alphabet letters between groups represent significant differences at *p* < 0.05.

**Figure 7:**
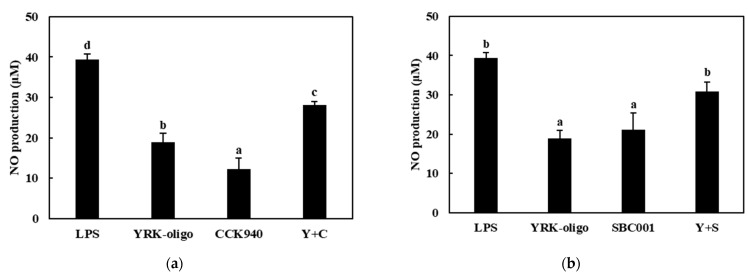

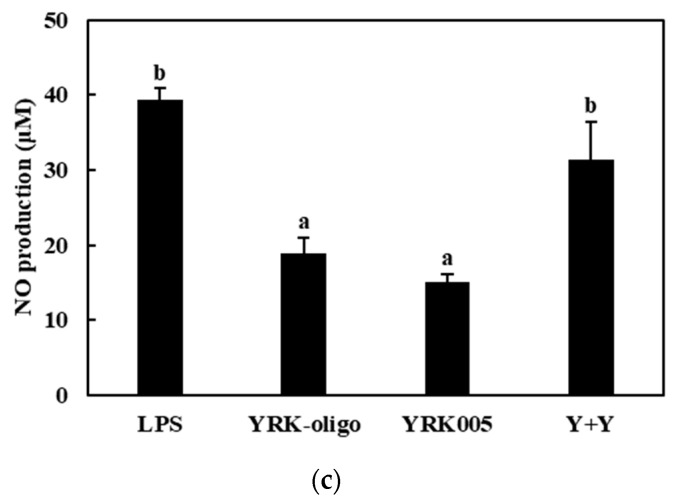
Effects of synbiotics containing YRK-oligosaccharides on nitric oxide production in RAW 264.7 macrophages. The combinations of probiotics and prebiotics used were: (**a**) YRK-oligosaccharides + *L. lactis* CCK940 (Y + C): 0.1 mg/mL of YRK-oligosaccharides and MOI 10 of *L. lactis* CCK940; (**b**) YRK-oligosaccharides + *L. lactis* SBC001 (Y + S): 0.1 mg/mL of YRK-oligosaccharides and MOI 20 of *L. lactis* SBC001; and (**c**) YRK-oligosaccharides + *W. cibaria* YRK005 (Y + Y): 0.1 mg/mL of YRK-oligosaccharides and MOI 20 of *W. cibaria* YRK005. Macrophages treated with 1 μg/mL of lipopolysaccharides (LPS) were used as the positive control sample. Different alphabet letters between groups represent significant differences at *p* < 0.05.

The nitric oxide production was assessed in the RAW 264.7 cells treated with the CCK-, SBC-, and YRK-oligosaccharides and with the *L. lactis* CCK940, *L. lactis* SBC001, and *W. cibaria* YRK005 strains at various concentrations and MOI values. The results showed that the macrophages treated with the CCK-, SBC-, and YRK-oligosaccharides produced 6.39-, 6.44-, and 18.89-μM concentrations of NO, respectively. In addition, the macrophages treated with the *L. lactis* CCK940, *L. lactis* SBC001, and *W. cibaria* YRK005 strains produced 12.29-, 13.69-, and 15.08-μM concentrations of NO, respectively. These results suggest that the CCK-, SBC-, and YRK-oligosaccharides and the *L. lactis* CCK940, *L. lactis* SBC001, and *W. cibaria* YRK005 strains potentially exert immunostimulatory activities when used alone (Figure 5, Figure 6 and Figure 7).

Next, the effects of the synbiotics containing combinations of one of the three probiotics (C, S, Y) and one of the three prebiotics (C, S, Y) on the NO production in the RAW 264.7 macrophages were investigated. The macrophages treated with the synbiotics containing CCK-oligosaccharides + *L. lactis* CCK940 (C + C), CCK-oligosaccharides + *L. lactis* SBC001 (C + S), and CCK-oligosaccharides + *W. cibaria* YRK005 (C + Y) produced 29.25-, 32.44-, and 31.47-μM concentrations of NO, respectively (Figure 5, Table 1). Notably, the concentrations of NO produced by the macrophages treated with these combinations were higher by 10.57, 12.36, and 10.00 μM than the sum of the NO concentrations produced by the macrophages treated individually with each corresponding probiotic and prebiotic. The macrophages treated with synbiotics containing SBC-oligosaccharides + *L. lactis* CCK940 (S+C), SBC-oligosaccharides + *L. lactis* SBC001 (S + S), and SBC-oligosaccharides + *W. cibaria* YRK005 (S + Y) produced 24.87-, 27.16-, and 25.72-μM concentrations of NO, respectively (Figure 6, Table 1). In addition, the concentrations of NO produced by the macrophages treated with these combinations were higher by 6.13, 7.03, and 4.19 μM than the sum of the NO concentrations produced by the macrophages treated individually with each corresponding probiotic and prebiotic. Finally, the macrophages treated with the synbiotics containing YRK-oligosaccharides + *L. lactis* CCK940 (Y + C), YRK-oligosaccharides + *L. lactis* SBC001 (Y + S), and YRK-oligosaccharides + *W. cibaria* YRK005 (Y + Y) produced 34.55-, 34.31-, and 35.86-μM concentrations of NO, respectively (Figure 7, Table 1). Moreover, the concentrations of NO produced by the macrophages treated with these combinations were higher by 3.36, 1.73, and 1.89 μM than the sum of the NO concentrations produced by the macrophages treated individually with each corresponding probiotic and prebiotic. These results suggest that the studied combinations of probiotics and prebiotics had a synergistic effect on the NO produced by the treated macrophages, which may have important implications for the significant immunostimulatory activities displayed by synbiotics containing these combinations.

#### 3.3.2. Quantitative Analysis of the Expressions of Cytokine Genes in Macrophages Treated with Synbiotics

**Figure 8:**
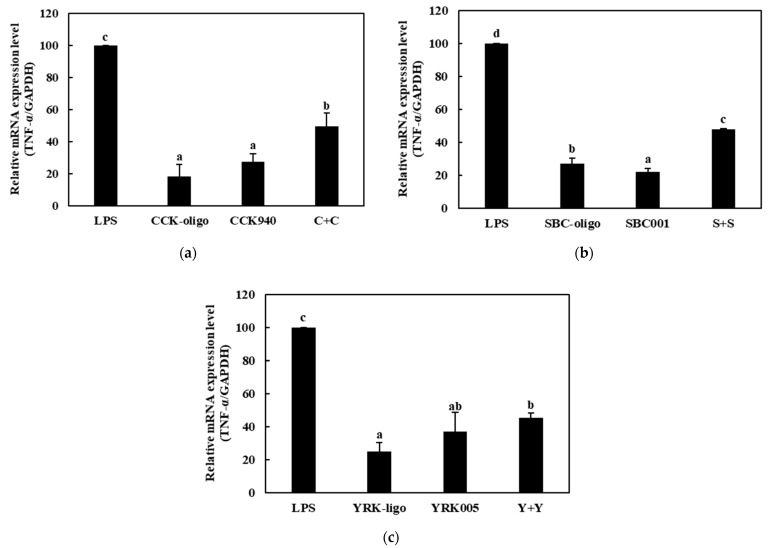
Effects of synbiotics on the mRNA-expression levels of the *TNF-α* gene in RAW 264.7 macrophages. The combinations of probiotics and prebiotics used were: (**a**) C + C: 0.25 mg/mL of CCK-oligosaccharides and MOI 10 of *L. lactis* CCK940; (**b**) S + S: 0.1 mg/mL of SBC-oligosaccharides and MOI 20 of *L. lactis* SBC001; and (**c**) Y + Y: 0.1 mg/mL of YRK-oligosaccharides and MOI 20 of *W. cibaria* YRK005. Macrophages treated with 1 μg/mL of lipopolysaccharides (LPS) were used as the positive control sample. Different alphabet letters between groups represent significant differences at *p* < 0.05.

**Figure 9:**
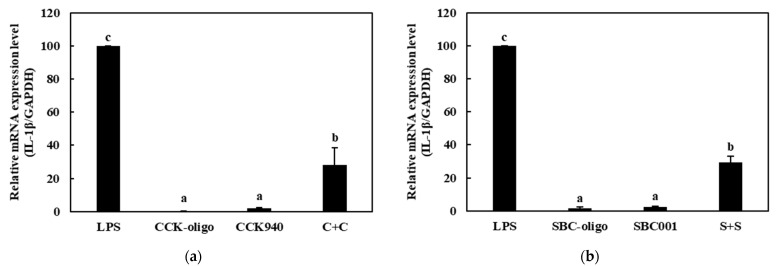

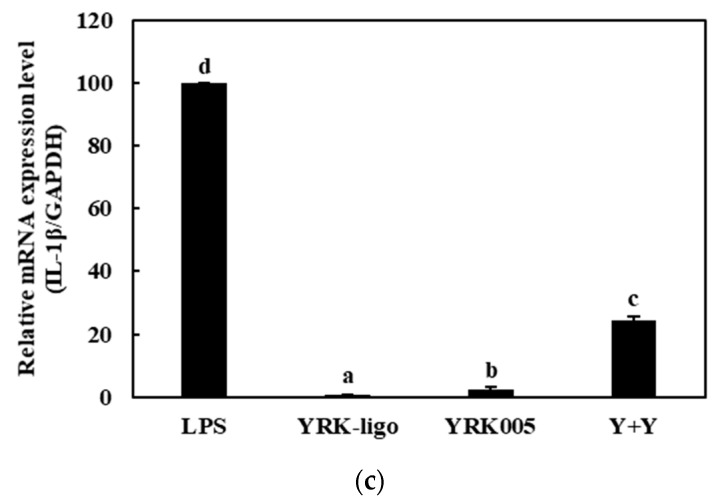
Effects of synbiotics on the mRNA expression levels of the *IL-1β* gene in RAW 264.7 macrophages. The combinations of probiotics and prebiotics used were: (**a**) C + C: 0.25 mg/mL of CCK-oligosaccharides and MOI 10 of *L. lactis* CCK940; (**b**) S + S: 0.1 mg/mL of SBC-oligosaccharides and MOI 20 of *L. lactis* SBC001; and (**c**) Y + Y: 0.1 mg/mL of YRK-oligosaccharides and MOI 20 of *W. cibaria* YRK005. Macrophages treated with 1 μg/mL of lipopolysaccharides (LPS) were used as the positive control sample. Different alphabet letters between groups represent significant differences at *p* < 0.05.

**Figure 10:**
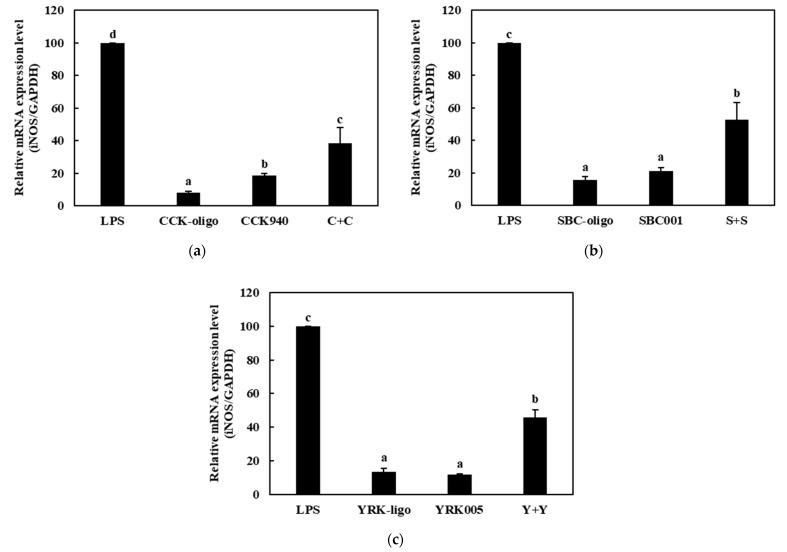
Effects of synbiotics on the mRNA expression levels of the *iNOS* gene in RAW 264.7 macrophages. The combinations of probiotics and prebiotics used were: (**a**) C + C: 0.25 mg/mL of CCK-oligosaccharides and MOI 10 of *L. lactis* CCK940; (**b**) S + S: 0.1 mg/mL of SBC-oligosaccharides and MOI 20 of *L. lactis* SBC001; and (**c**) Y + Y: 0.1 mg/mL of YRK-oligosaccharides and MOI 20 of *W. cibaria* YRK005. Macrophages treated with 1 μg/mL of lipopolysaccharides (LPS) were used as the positive control sample. Different alphabet letters between groups represent significant differences at *p* < 0.05.

**Figure 11:**
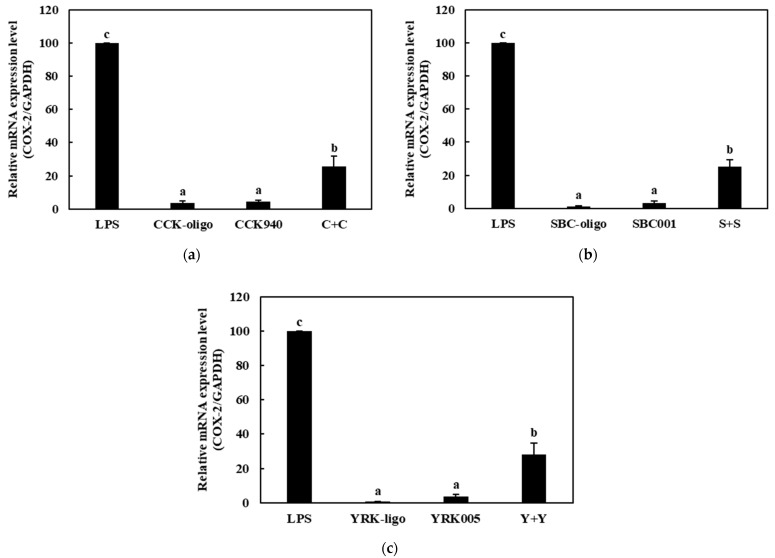
Effects of synbiotics on the mRNA expression levels of the *COX-2* gene in RAW 264.7 macrophages. The combinations of probiotics and prebiotics used were: (**a**) C + C: 0.25 mg/mL of CCK-oligosaccharides and MOI 10 of *L. lactis* CCK940; (**b**) S + S: 0.1 mg/mL of SBC-oligosaccharides and MOI 20 of *L. lactis* SBC001; and (**c**) Y + Y: 0.1 mg/mL of YRK-oligosaccharides and MOI 20 of *W. cibaria* YRK005. Macrophages treated with 1 μg/mL of lipopolysaccharides (LPS) were used as the positive control sample. Different alphabet letters between groups represent significant differences at *p* < 0.05.

We investigated the immunostimulatory effects of the three homologous combinations of probiotics and prebiotics, i.e., C + C, S + S, and Y + Y, on the RAW 264.7 cells by determining the mRNA expression levels of the cytokine genes in the treated cells. The RAW 264.7 cells were treated with 0.25-, 0.1-, and 0.1-mg/mL concentrations of the CCK-, SBC-, and YRK-oligosaccharides, respectively, and 10-, 20-, and 20-MOI concentrations of the *L. lactis* CCK940, *L. lactis* SBC001, and *W. cibaria* YRK005 strains, respectively. The macrophages treated with 1 μg/mL of lipopolysaccharides (LPS) were used as the positive control sample. The expression levels of the cytokine genes (*TNF-α*, *IL-1β*, *iNOS*, and *COX-2*) in the macrophages treated with the three synbiotics are shown in Figure 8, Figure 9, Figure 10 and Figure 11. The results show that the expression levels of the *IL-1β*, *iNOS*, and *COX-2* genes increased significantly in the macrophages treated with the three synbiotics. Although the mRNA-expression levels of the *TNF-α* gene also increased, this increase was lower than those in the other cytokine genes. These findings suggest that synbiotics may play a role in enhancing the immunostimulatory activities of macrophages. 

#### 3.3.3. Quantitative Analysis of the Expression of the MAPK-Pathway Proteins in Macrophages Treated with Synbiotics

**Figure 12:**
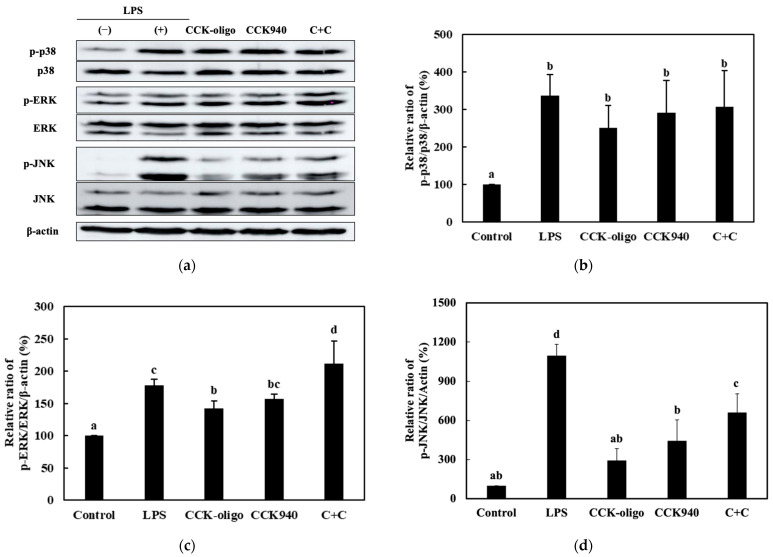
Effects of C + C synbiotic treatment on MAPK-signaling pathway in RAW 264.7 macrophages. (**a**) Western blot reveals the expression of MAPK-pathway proteins in macrophages treated with the C + C synbiotic, i.e., 0.25 mg/mL of CCK-oligosaccharides and MOI 10 of *L. lactis* CCK940, and with the corresponding prebiotics and probiotics alone. Graphs depict the relative ratios of (**b**) p-p38/p38, (**c**) p-ERK/ERK, and (**d**) p-JNK/JNK in macrophages treated with the C + C synbiotic and with CCK-oligosaccharides and *L. lactis* CCK940 alone. Western blot is representative of three independent experiments. Macrophages treated with 1 μg/mL of lipopolysaccharides (LPS) were used as the positive control sample. Different alphabet letters between groups represent significant differences at *p* < 0.05.

**Figure 13:**
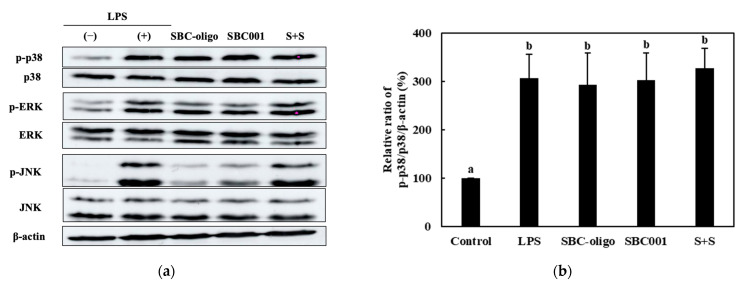

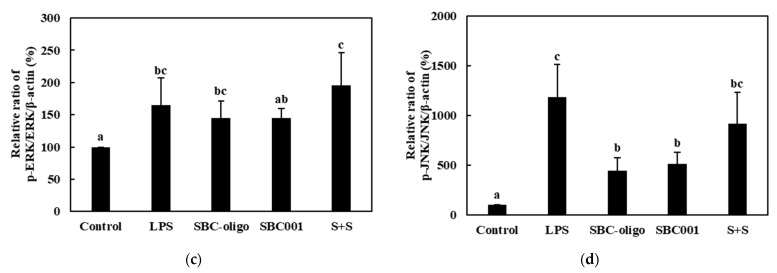
Effects of S + S synbiotic treatment on MAPK-signaling pathway in RAW 264.7 macrophages. (**a**) Western blot reveals the expression of MAPK-pathway proteins in macrophages treated with the S + S synbiotic, i.e., 0.1 mg/mL of SBC-oligosaccharides and MOI 20 of *L. lactis* SBC001, and with the corresponding prebiotics and probiotics alone. Graphs depict the relative ratios of (**b**) p-p38/p38, (**c**) p-ERK/ERK, and (**d**) p-JNK/JNK in macrophages treated with the S + S synbiotic and with SBC-oligosaccharides and *L. lactis* SBC001 alone. Western blot is representative of three independent experiments. Macrophages treated with 1 μg/mL of lipopolysaccharides (LPS) were used as the positive control sample. Different alphabet letters between groups represent significant differences at *p* < 0.05.

**Figure 14:**
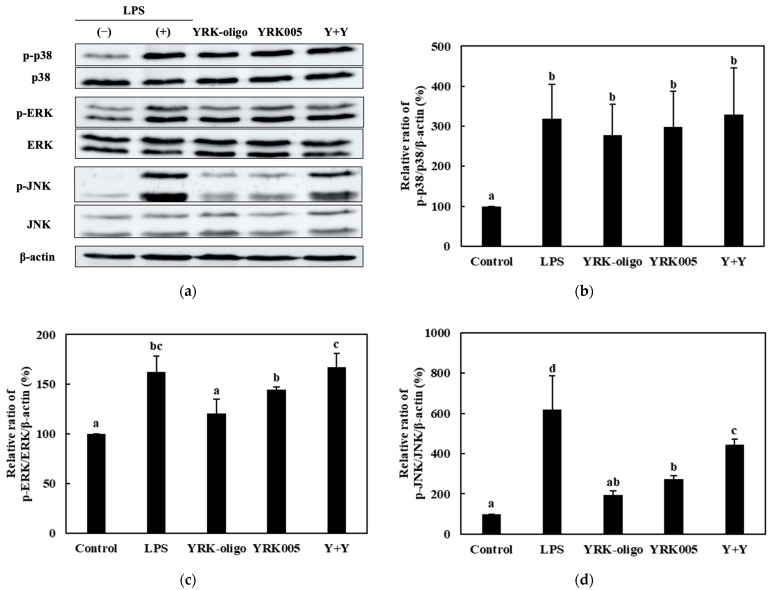
Effects of Y + Y synbiotic treatment on MAPK-signaling pathway in RAW 264.7 macrophages. (**a**) Western blot reveals the expression of MAPK-pathway proteins in macrophages treated with the Y + Y synbiotic, i.e., 0.1 mg/mL of YRK-oligosaccharides and MOI 20 of *W. cibaria* YRK005, and with the corresponding prebiotics and probiotics alone. Graphs depict the relative ratios of (**b**) p-p38/p38, (**c**) p-ERK/ERK, and (**d**) p-JNK/JNK in macrophages treated with the Y + Y synbiotic and with YRK-oligosaccharides and *W. cibaria* YRK005 alone. Western blot is representative of three independent experiments. Macrophages treated with 1 μg/mL of lipopolysaccharides (LPS) were used as the positive control sample. Different alphabet letters between groups represent significant differences at *p* < 0.05.

**Figure 15:**
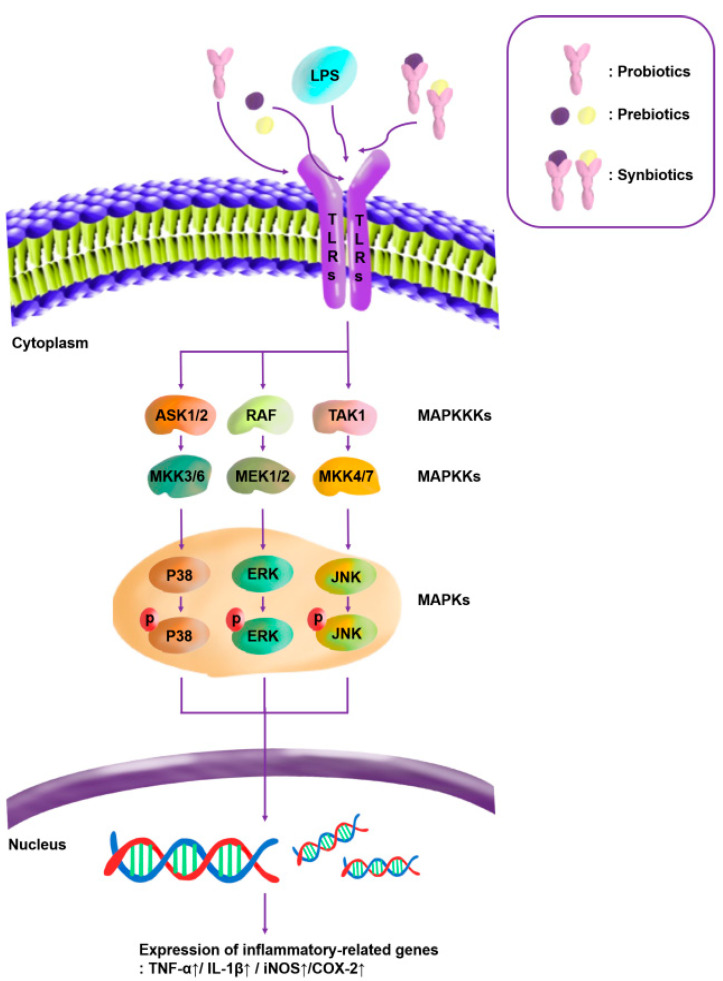
Diagram depicting the predicted immunomodulatory signaling mechanism underlying the effects of pro-/pre-/synbiotics on RAW 264.7 macrophages. TLRs, Toll-like receptors; ASK, apoptosis-signal-regulating kinase; MEK, mitogen-activated extracellular-signal-regulated kinase; MKK, mitogen-activated protein kinase kinase; RAF, rapidly accelerated fibrosarcoma; TAK, transforming growth factor-β activated kinase; and *COX-2*, cyclooxygenase-2 gene.

Among the nine synbiotic preparations used in this study, we investigated the effects of the three homologous probiotic + prebiotic combinations, i.e., C + C, S + S, and Y + Y, on the ratios of p-p38/p-38, p-ERK/ERK, and p-JNK/JNK. The RAW 264.7 cells were treated with 0.25-, 0.1-, and 0.1-mg/mL concentrations of the CCK-, SBC-, and YRK-oligosaccharides, respectively, and 10-, 20-, and 20-MOI concentrations of the *L. lactis* CCK940, *L. lactis* SBC001, and *W. cibaria* YRK005 strains, respectively. The p38/p-38, p-ERK/ERK, and p-JNK/JNK ratios are shown in Figure 12, Figure 13, Figure 14 and Figure 15. The results show that the macrophages treated with the three synbiotics exhibited a synergistic enhancement in their ratios of p-JNK/JNK compared to the macrophages treated with the corresponding probiotics and prebiotics alone. Of the three combinations, the treatment with C + C resulted in the most significant increase in the ratio of p-JNK/JNK (Figure 12d). The increase in the p-p38/p38 ratios was not significant in the macrophages treated with any of the three synbiotics. The increases in the p-ERK/ERK ratios were not significantly different between the three combinations; however, the C + C combination showed the highest gain. These findings suggest that the synbiotics prepared in this study modulate the immunostimulatory activity of macrophages via the MAPK-signaling pathway.

## 4. Discussion

Lactic acid bacteria exist in the human digestive tract and oral cavity [37]. Acid and bile tolerances are the two fundamental properties that determine the ability of probiotic microorganisms to survive in the acidic environment of the upper gastrointestinal tract and the presence of bile salts in the intestine. For use as probiotics, the survival of *L. lactis* CCK940, *L. lactis* SBC001, and *W. cibaria* YRK005 strains was confirmed under acidic conditions and with good viability [38]. Bacteria with acid tolerance can survive in the presence of the gastric acid secreted by the stomach [39]. According to a previous study, *L. lactis* SPK021 and *L. lactis* SG-005 strains showed acid tolerances of 58.8% and 28.4%, respectively, whereas the strains *W. cibaria* SPK014, *W. cibaria* Bro008, and *W. cibaria* Bro014 exhibited acid tolerances of 40.9, 39.7, and 14.9%, respectively [35].

The standard range of human bile concentrations is 0.3–0.5%. Therefore, bile tolerance is a critical probiotic characteristic that enables lactic acid bacteria to survive in the human small intestine. Many studies demonstrated that numerous strains of lactic acid bacteria can survive well under these conditions, indicating the possibility of restoring the initial number of cells that pass through the small intestine. Consequently, it was suggested that strains that survive under these conditions are resistant to stomach and intestinal environments [35,39]. A previous study reported that *Lactobacillus acidophilus* and *Lactiplantibacillus plantarum* species experienced a reduction in log CFU of 2.47 and 2.71 during growth on media supplemented with 0.3% Oxgall media for 24 h [40]. Based on these results, the bacterial strains used in this study were observed to have a bile resistance superior to those of other general lactic acid bacterial strains.

As the ability to combat oxidative stress decreases with age, the cellular environment can become increasingly oxidative, eventually leading to severe health conditions, such as Alzheimer’s disease and diabetes [41]. Consequently, efforts aimed at the development and utilization of effective natural antioxidants to protect the human body from reactive oxygen species (ROS) and delay the progression of various chronic diseases have gained in significance. A previous study reported the DPPH radical-scavenging activities of different lactic acid bacterial strains. Specifically, the *Lactiplantibacillus paraplantarum* SC61, *L. citreum* S. Pum19, and *Pediococcus pentosaceus* SC28 strains exhibited 51.1%, 32.7%, and 32.6% DPPH radical-scavenging activities, respectively [42]. Based on these results, the three lactic acid bacterial strains used in this study demonstrated higher antioxidant abilities than those of the lactic acid bacteria used in previous studies.

The immune-related effects of probiotic strains appear to be mediated by temporary or long-term colonization through adhesion and aggregation without invasion [43]. Thus, the intestinal surface available for bacterial cell adhesion and the clustering ability of these strains may be necessary preconditions for probiotics to exert beneficial effects on the large intestine. In the present study, experiments were conducted to assess the adhesion ability of lactic acid bacteria to intestinal cells using HT-29 cells, a commonly used method to confirm the probiotic functions of these bacteria. According to a previous study, the adhesion abilities of 10 evaluated *Lactobacillus* strains to intestinal cells ranged from 20% to 80% [39]. According to these results, *L. lactis* CCK940, *L. lactis* SBC001, and *W. cibaria* YRK005 strains exhibited a good ability to adhere to intestinal cells.

We conducted various assays, including a cell-cytotoxicity assay, a NO assay, an evaluation of the expressions of pro-inflammatory cytokine genes, and an assessment of the expression levels of MAPK-pathway proteins, to evaluate the immunomodulatory effects of the probiotics on the macrophages. The cell viability was evaluated using the EZ-Cytox method, in which water-soluble tetrazolium salt was used to form formazan dye at bacterial concentrations ranging from 0 to 500, in order to determine whether the three lactic acid bacteria conferred cytotoxicity on the macrophages [44]. A previous study demonstrated that NO production increased in a ratio-dependent manner; thus, it increased in line with the increases in the ratio of lactic acid bacteria to macrophages [45]. This finding was consistent with those of several other studies, which reported the immunostimulatory effects of certain lactobacilli and their ability to stimulate macrophages to induce NO production based on the ratio of bacterial cells to macrophages. These results suggest that the three lactic acid bacterial strains can be used as probiotics with immunostimulatory properties. Our previous study showed that *L. lactis* SBC001 had anti-inflammatory effects on RAW 264.7 cells [32]. Combined with the results of that study, this study revealed that *L. lactis* SBC001 has both immunostimulatory and anti-inflammatory activities. The RAW 264.7 macrophages, which present antigens and phagocytose foreign substances, were used in this study to measure the immune activities of various substances (e.g., bacterial oligosaccharides) [29,46]. The inflammatory response involves NO, a small, diffusible, short-lived molecule predominantly synthesized endogenously by the *iNOS* gene as both a regulator and a mediator [47]. Nitric oxide has been implicated in the macrophage-mediated eradication of invading pathogens, owing to its ability to act as an effector molecule [48]. Therefore, in this study, the RAW 264.7 macrophages were treated with the same concentration of each probiotic strain, and each probiotic strain produced NO in a concentration-dependent manner.

Macrophages such as the RAW 264.7 used in this study release cytokines through endocrine systems that can affect the immune response and inflammatory control [29]. The TNF-α and IL-1β cytokines produced by stimulated macrophages play critical roles in the innate immune system [49]. Inflammation intensifies the phagocytosis of stimulated macrophages and the secretion of cytokines, such as TNF-α and IL-1β, kills pathogens. Moreover, stimulated macrophages protect against foreign-factor invasions that cause inflammation by secreting ROS, NO, and other mediators against pathogenic microorganisms [47]. Initially identified as a serum factor capable of inducing tumor necrosis, TNF-α is now recognized as a potent immunomodulatory cytokine [50], and IL-1β, an inflammatory cytokine that directly interacts with vascular endothelial cells and enhances the production of proangiogenic factors through paracrine regulation, can regulate angiogenesis [51]. The expression levels of the *TNF-α* gene of macrophages treated with *Levilactobacillus brevis* KU15147 and *Lev. brevis* KU15154 strains were lower than those in macrophages induced with 10.0 ng/mL LPS [52]. Compared to the results reported in these studies, the three strains used in this study were observed to express the *TNF-α* gene effectively. The COX-2 protein is induced transiently by pro-inflammatory cytokines, growth factors, bacterial toxins, and tumor promoters, and it is detected only in specific types of tissue [53]. The MAPKs are a group of protein kinases with conserved regulatory functions ranging from unicellular to complex organisms. Protein kinases covalently attach phosphate groups to threonine, serine, and tyrosine side chains in specific cellular proteins. These phosphorylated proteins can control interactions with other molecules, their enzymatic activity and location in cells, and their tendency to be decomposed by proteases [54]. 

The mammalian immune system has developed innate and adaptive immunity as its principal function, which protects organisms from foreign pathogens [55]. Activated MAPKs, including p38, ERK, and JNK, play significant roles in innate and adaptive immunity [55,56]. This study showed that the three strains used effectively activated the JNK signaling pathway. The *L. lactis* CCK940 and *L. lactis* SBC001 strains stimulated the p38- and ERK-signaling pathways, whereas the *W. cibaria* YRK005 strain did not. The evaluation of the expressions of various pro-inflammatory-inducing proteins in this study confirmed that the three lactic strains demonstrated immunomodulatory activities. Therefore, the *L. lactis* CCK940, *L. lactis* SBC001, and *W. cibaria* YRK005 strains could be potential probiotic strains.

The *L. lactis* CCK940, *L. lactis* SBC001, and *W. cibaria* YRK005 strains have glycosyltransferases (GTs), such as maltose phosphorylase and dextransucrase, which produce oligosaccharides [57,58]. These strains also have glycoside hydrolase (GH) genes, which utilize oligosaccharides, such as β-galactosidase and α-glucosidase (EC 3.2.1.20). The *L. lactis* CCK940 and *L. lactis* SBC001 strains have α-galactosidase enzymes, which hydrolyze raffinose or stachyose into glucose and sucrose. The three lactic acid bacteria used in this study also contain the dextransucrase enzyme, which catalyzes the synthesis of dextran using sucrose and maltose as donor and receptor molecules, respectively. Additionally, the three strains have the maltose phosphorylase enzyme, which catalyzes the phosphorolysis of maltose into β-D-glucose-1-phosphate and glucose, and α-glucosidase, which hydrolyzes starch or disaccharides into glucose. Furthermore, these strains have the β-galactosidase enzyme, which hydrolyzes lactose into glucose and galactose, as well as hydrolyzing a broad range of GOS (including oligosaccharides with 3–6 DP) [6]. An in silico analysis suggested that the three lactic acid bacterial strains contained enzymes synthesizing oligosaccharides [57,58].

The CE1 family includes diacylglycerol *O*-acyltransferases (EC 2.3.1.20), carboxylesterases (EC 3.1.1.1), and acetyl xylan esterase (EC 3.1.1.72) [59]. The GH1, GH2, and GH42 families include β-galactosidase (EC 3.2.1.23), and they synthesize GOS using lactose as a substrate [60]. The GH13 and GH65 families contain sucrose phosphorylase (EC 2.4.1.7) and maltose phosphorylase (EC 2.4.1.8), respectively, which synthesize oligosaccharides [61,62]. The GT2 and GT4 families include various synthases, such as cellulose synthase (EC 2.4.1.12) and sucrose synthase [63]. Previous studies reported that the *L. lactis* NZ6009 and *W. cibaria* 37 strains also possess the gene encoding β-galactosidase [64,65]. 

Synbiotics, which are combinations of probiotics and prebiotics, are increasingly recognized for their potential to improve health and prevent diseases. Of the various types of synbiotics, those composed of different prebiotics and probiotics have shown promising results regarding their anti-inflammatory and immune-enhancing effects. The ingestion of 8g/d scFOS for 12 days by healthy elderly individuals increased fecal bifidobacteria and produced significant changes in cholesterol metabolism, with potentially protective effects against colon cancer [66]. Another study found that a synbiotic diet containing *L. casei* ASCC 292, FOS, and maltodextrin lowered serum cholesterol and triglyceride levels, increased serum high-density-lipoprotein-cholesterol levels, and produced a healthier bowel microbial population without lactobacilli translocation [67]. Additionally, a study investigating the effects on XOS of corn cobs combined with *L. plantarum* found that the synbiotic combination enhanced the growth rate and density of the *L. plantarum* S2 cells, increased the antimicrobial activities against gastrointestinal pathogens, and exhibited significant antioxidant activities [68]. These findings indicate that specific prebiotics and probiotics used together may enhance the beneficial effects of synbiotics and provide potential health benefits by encouraging anti-inflammatory and immune-enhancing responses.

In addition to their immunomodulatory properties, our previous research showed that the three lactic acid bacteria used in this study have prebiotic activities that encourage the growth of probiotics. The *Leu. lactis* CCK940 was superior to fructo-oligosaccharides, typical commercial prebiotics, in its growth-promoting activities against *Lacticaseibacillus casei*, *L. pentosus*, *Lactiplantibacillus plantarum*, *W. cibaria*, *Bifidobacterim animalis,* and *Saccharomyces cerevisiae* [11], while *L. lactis* SBC001 was superior to fructo-oligosaccharides in terms of growth-promoting activities against *L. plantarum*, *Lacticaseibacillus paracasei*, *L. johnsonii*, *Leuconostoc mesenteroides*, *Lacticaseibacillus rhamnosus,* and *S. cerevisiae* [32]. Furthermore, *W. cibaria* YRK005 was also superior to fructo-oligosaccharides in terms of growth-promoting activities against *B. adolescentis*, *L. acidophilus*, *L. lactis,* and *L. pentosus* [33].

In this study, we evaluated the immunostimulatory activities of synbiotics by measuring the NO production, the gene expressions of pro-inflammatory cytokines, and the protein-expression levels of the MAPK-pathway proteins. The combination of the CCK-oligosaccharides and the *L. lactis* SBC001 strain led to the highest increase in NO production, with a gain of 12.36 μM over the sum of the NO concentrations produced when the CCK-oligosaccharides and *L. lactis* SBC001 were used alone. In contrast, the combination of the YRK-oligosaccharides and the *L. lactis* SBC001 strain led to the smallest increase, with a gain of 1.73 μM over the sum of the NO concentrations produced when the YRK-oligosaccharides and the *L. lactis* SBC001 strain were used alone. The gene-expression levels of IL-1β were significantly increased in all the synbiotics, as measured by the qPCR. The Western blotting confirmed that the ratios of p-JNK/JNK showed the highest increases in response to the synergistic effects of all the probiotic + prebiotic combinations used in this study. The immunomodulatory signaling mechanism underlying the effects of the pro-/pre-/synbiotics on the RAW 264.7 macrophages is illustrated in Figure 15.

Furthermore, in this study, we investigated the immunostimulatory activity of synbiotics prepared by combining a probiotic strain with prebiotic oligosaccharides. The probiotic strain used produces oligosaccharides that act as prebiotics. This means that they can use the oligosaccharides they produce as prebiotics to increase their own growth. This study confirmed this observation, which is significant because such a study has never been attempted previously. If oligosaccharide-producing probiotic strains are cultured under conditions suitable for the production of oligosaccharides, it is expected that the growth of the bacteria can be increased by using the oligosaccharides they produce as prebiotics. If these conditions are met in the human intestinal tract, the oligosaccharides produced by these strains should encourage the growth of probiotic strains in the gut.

## 5. Conclusions

We confirmed the probiotic characteristics and immune activities of three lactic acid bacterial strains producing the following prebiotic oligosaccharides: *L. lactis* CCK940, *L. lactis* SBC001, and *W. cibaria* YRK005. The synbiotics were developed using each probiotic strain and its corresponding prebiotic oligosaccharide, showing high immunostimulatory activity. This method allows the use of probiotic-synthesized prebiotic oligosaccharides, leading to synergistic immunomodulatory effects when administered. These synbiotic preparations are recommended for the manufacturing of health-promoting supplements.

## Figures and Tables

**Table 1:** Nine synbiotic preparations representing combinations of prebiotic oligosaccharides and probiotic lactic acid bacteria.

Sample	Synbiotic Preparation
C + C	CCK-oligosaccharides + *Leuconostoc lactis* CCK940
C + S	CCK-oligosaccharides + *L. lactis* SBC001
C + Y	CCK-oligosaccharides + *Weissella cibaria* YRK005
S + C	SBC-oligosaccharides + *L. lactis* CCK940
S + S	SBC-oligosaccharides + *L. lactis* SBC001
S + Y	SBC-oligosaccharides + *W. cibaria* YRK005
Y + C	YRK-oligosaccharides + *L. lactis* CCK940
Y + S	YRK-oligosaccharides + *L. lactis* SBC001
Y + Y	YRK-oligosaccharides + *W. cibaria* YRK005

**Table 2:** Primer sequences for real-time quantitative polymerase chain reaction (qPCR).

Gene		Primer Sequence
*GAPDH*	Forward Reverse	5′-ATC CCA TCA CCA TCT TCC AG-3′ 5′-CCT GCT TCA CCA CCT TCT TG-3′
*TNF-α*	Forward Reverse	5′-ATG AGC ACA GAA AGC ATG ATC CG-3′ 5′-CCA AAG TAG ACC TGC CCG GAC TC-3′
*IL-1β*	Forward Reverse	5′-ATG GCA ACT GTT CCT GAA CTC AACT-3′ 5′-CAG GAC AGG TAT AGA TTC TTT CCT T-3′
*iNOS*	Forward Reverse	5′-AAT GGC AAC ATC AGG TCG GCC ATC ACT-3′ 5′-GCT GTG TGT CAC AGA AGT CTC GAA CTC-3′

Abbreviations: GAPDH, glyceraldehyde-3-phosphate dehydrogenase; TNF-α, tissue necrosis factor-α; IL-1β, interleukin-1β; and iNOS, inducible nitric oxide synthase.

**Table 3:** Acid and bile tolerances and adhesion ability of *L. lactis* CCK940, *L. lactis* SBC001, and *W. cibaria* YRK005 strains.

Strain	Acid Tolerance (%)	Bile Tolerance (%)	Adhesion Ability (%)
*Leuconostoc lactis* CCK940	87.2 ± 3.9 ^b^	75.9 ± 1.3 ^a^	54.2 ± 11.1 ^a^
*L. lactis* SBC001	52.3 ± 3.3 ^a^	81.9 ± 0.3 ^b^	55.7 ± 10.7 ^a^
*Weissella cibaria* YRK005	54.0 ± 3.4 ^a^	82.4 ± 1.9 ^b^	67.6 ± 5.4 ^a^

Data are expressed as means ± standard deviation of three independent experiments. Different letters show significant differences between groups at *p* < 0.05 (n ≥ 3).

**Table 4:** DPPH radical-scavenging activities of probiotics.

Strain	Antioxidant Activity (%)
*Leuconostoc lactis* CCK940	56.8 ± 3.1 ^a^
*L. lactis* SBC001	67.1 ± 2.2 ^b^
*Weissella cibaria* YRK005	64.5 ± 3.2 ^b^

Data are expressed as means ± standard deviation of three independent experiments. Different letters show significant differences between groups at *p* < 0.05 (n ≥ 3).

## Data Availability

Not applicable.
